# Associations between AI literacy and learning engagement in smart classrooms: the chain mediating roles of self-efficacy and learning satisfaction

**DOI:** 10.3389/fpsyg.2026.1931835

**Published:** 2026-09-23

**Authors:** Zhidan Liao, Xiujie Zhou, Danni Dai, Jianchao Ni

**Affiliations:** 1 Minnan Normal University, Zhangzhou, Fujian, China; 2 Xiamen University of Technology, School of Film Television and Communication, Xiamen, Fujian, China; 3 Office of Student Affairs, Xiamen University, Xiamen, Fujian, China; 4 Institute of Education, Xiamen University, Xiamen, Fujian, China; 5 School of Marxism, Xiamen University, Xiamen, Fujian, China

**Keywords:** AI literacy, chain mediation, learning engagement, learning satisfaction, self-efficacy, smart classroom

## Abstract

**Background:**

With the increasing integration of artificial intelligence (AI) into education, smart classrooms have become an important component of higher education's digital transformation. College students' learning engagement is an important indicator for understanding learning experiences in smart classroom environments. However, as a key capability for adapting to AI-supported learning, the psychological pathways through which AI literacy is associated with learning engagement remain insufficiently understood. This study constructed a chain mediation model to examine the association between AI literacy and college students' learning engagement in smart classrooms, with self-efficacy and learning satisfaction as potential mediators.

**Methods:**

This study employed an exploratory cross-sectional design. A total of 2,751 valid questionnaires were collected from 75 colleges and universities in China using a convenience sampling method. Data were collected using the AI Literacy Scale, Learning Engagement Scale, Self-Efficacy Scale, and Learning Satisfaction Scale. Confirmatory factor analysis (CFA), correlation analysis, and a preliminary single-factor assessment of potential common method bias were conducted using SPSS 26 and AMOS 26. Percentile bootstrap analysis using 5,000 resamples in the PROCESS macro was conducted to test the chain mediating roles of self-efficacy and learning satisfaction.

**Results:**

(1) AI literacy was significantly and positively associated with learning engagement in smart classrooms (total effect, *B* = 0.899, *p* < 0.001; direct effect after including the mediators, *B* = 0.304, *p* < 0.001); (2) self-efficacy mediated the association between AI literacy and learning engagement [indirect effect = 0.084, 95% CI = (0.060, 0.108)]; (3) learning satisfaction mediated the association between AI literacy and learning engagement [indirect effect = 0.427, 95% CI = (0.380, 0.476)]; and (4) the serial indirect path through self-efficacy and learning satisfaction was significant [indirect effect = 0.085, 95% CI = (0.064, 0.108)]. The total indirect effect was B = 0.595 [95% CI = (0.544, 0.644)] and accounted for 66.185% of the total effect.

**Conclusion:**

AI literacy was directly associated with learning engagement and was also indirectly associated with learning engagement through self-efficacy and learning satisfaction. These findings provide preliminary cross-sectional evidence for understanding the cognitive–affective pathways linking AI literacy and learning engagement in smart classroom contexts. Rather than establishing causal effects, the study offers preliminary evidence that students' self-efficacy and learning satisfaction may be important psychological factors in AI-supported learning environments.

## 1 Introduction

With the rapid development of artificial intelligence (AI) technology, smart classrooms, as an important form of digital transformation in education, are profoundly transforming the teaching ecosystem of higher education. As the primary participants in these environments, college students' engagement and behavioral adaptability are now decisive factors in determining teaching effectiveness. AI literacy, defined as students' comprehensive ability to understand and use AI tools, has been identified as a key factor for effective learning in technology-driven learning environments (Ng, 2012; Long and Magerko, 2020). Students with higher AI literacy are more likely to proactively adapt to teaching systems, and demonstrate greater learning engagement and behavioral participation in smart classrooms (Henrie et al., 2015).

While existing studies emphasize the importance of AI literacy for academic adaptability, they have largely examined surface-level associations without clarifying the internal mechanisms driving such behavioral outcomes. However, this pathway is not straightforward and may involve a series of mediating mechanisms. In fact, with the increasing integration of artificial intelligence into teaching and learning, several practical and pedagogical challenges have emerged. As artificial intelligence becomes increasingly integrated into teaching and learning, students may differ in their ability to understand, evaluate, and appropriately use AI-supported learning tools, which may shape their confidence, emotional experience, and engagement in smart classroom learning. These challenges highlight the need to further explore how psychological and emotional mechanisms mediate the relationship between AI use and students' engagement and satisfaction. Recent work locates AI-supported higher education within a broader shift toward digital and AI-integrated pedagogy. Faculty-level studies have examined digital transformation and teaching competence (Younas et al., 2025a), while an intelligent-TPACK model emphasizes the integration of technological, pedagogical, content, and ethical knowledge (Younas and Noor, 2026). Although these studies clarify how institutions and educators may integrate AI, in-depth investigation of student-level psychological processes—particularly the potential pathways linking AI literacy, self-efficacy, learning satisfaction, and engagement—remains comparatively limited. Further research is therefore needed to clarify these interrelationships.

Existing research has revealed that students' technical literacy not only affects their behavioral responses but also influences their self-efficacy at the cognitive level (Bandura, 1997; Prior et al., 2016). These findings suggest that cognitive variables such as self-efficacy may serve as critical pathways through which AI literacy influences students' learning behaviors. In AI-enabled teaching environments, whether students possess sufficient operational confidence and technical understanding will directly impact their ability to effectively manage the learning process, thereby influencing the maintenance of learning motivation and the development of learning behaviors.

Equally important, students' AI literacy may also indirectly impact their learning satisfaction, as recent studies have highlighted that digital technology use and AI-related competencies enhance learning engagement and emotional experiences in online contexts (Shao and Kang, 2022; Du et al., 2024; Zhou and Peng, 2025). When students are proficient in using AI tools in smart classrooms and smoothly complete learning tasks, they are more likely to experience positive emotions and value, thereby increasing their overall satisfaction with the class (Kuo et al., 2014). Learning satisfaction, as a psychological variable, has been widely demonstrated to be an important factor influencing learning persistence and behavioral engagement (Fredricks et al., 2004; Kuo et al., 2013). Together, these studies underline the importance of both cognitive and affective factors—namely, self-efficacy and learning satisfaction—as mediators in the relationship between AI literacy and engagement. Recent empirical work (Wang and Hui, 2024) has further demonstrated that in online English-learning contexts, learners with higher academic buoyancy tend to demonstrate greater engagement, with this relationship being mediated by lower levels of anxiety and boredom and higher levels of enjoyment. This evidence underscores that, beyond learning satisfaction *per se*, the dynamic interplay of emotional and motivational factors plays a crucial role in sustaining engagement in digital learning environments.

Therefore, AI literacy may not only influence students' learning behaviors in smart classrooms by enhancing their self-efficacy, but also indirectly affect their learning engagement by improving their classroom satisfaction. The interaction between AI literacy, self-efficacy, and learning satisfaction constitutes a key path to understanding college students' learning engagement in smart classrooms. Clarifying this association may provide preliminary evidence for understanding students' learning experiences in AI-supported smart classroom contexts.

Grounded in Expectancy-Value Theory (EVT), this study investigates the pathways through which college students' AI literacy influences their learning engagement in smart classrooms. EVT was developed in the 1980's, and is widely used to explain achievement-related behaviors such as motivation, behavior selection, and sustained engagement in educational settings (Eccles and Wigfield, 2002). This theory holds that whether individuals engage in a learning activity depends primarily on their expectation of success and their judgment of the task value, which together determine their behavioral performance (Wigfield and Eccles, 2000). This theory provides a robust theoretical framework for the relationship between AI literacy, self-efficacy, learning satisfaction, and learning engagement.

Within the theoretical framework of EVT, an individual's background variables (such as skill level, educational resources, cultural capital, etc.,) significantly influence the formation and change of their motivational system (Eccles and Wigfield, 2002). In the highly technological learning environment of smart classrooms, AI literacy, as students' ability to understand, operate, and evaluate AI technology, constitutes an important foundation for their adaptation to and mastery of learning systems. Students with good AI literacy are more likely to understand the diverse information structures and AI-assisted tools in smart classrooms, thereby enhancing their confidence and ability to complete tasks. Thus, AI literacy can be regarded as a key background variable affecting “success expectations,” directly impacting students' self-efficacy (Bandura, 1997; Prior et al., 2016). EVT further points out that self-efficacy is the core manifestation of “expectancy for success” and is an individual's self-assessment of ability when facing a specific learning task. In smart classrooms, students with a strong sense of self-efficacy are more likely to proactively address the challenges posed by AI tools and build a sense of control and confidence through continuous feedback.

In parallel, EVT also emphasizes the role of individual value judgments of tasks in the formation of behavioral motivation. These value judgments include dimensions such as intrinsic value, utility value, attainment value, and cost (Wigfield and Eccles, 2000). Learning satisfaction in this study reflects students' comprehensive emotional and cognitive evaluations of smart classroom learning activities, covering aspects such as the interest in the learning content, the practicality of the course, and personal identification. It essentially reflects the task value composition in EVT, and this interpretation follows the recent extensions of Expectancy–Value Theory, which recognize that affective evaluations such as learning satisfaction can serve as contextual manifestations of perceived task value in technology-enhanced learning environments (Eccles and Wigfield, 2020; Barron and Hulleman, 2015), thereby linking students' emotional and cognitive evaluations to their engagement processes within the EVT framework.

Importantly, EVT highlights that expectancy and value do not function in isolation; rather, they interactively shape individuals' motivational outcomes. Even when students feel confident in their ability to succeed (high expectancy), they may not be fully engaged unless they also perceive the task as worthwhile (high value), and vice versa. Therefore, it is the combined effect of these two dimensions that most strongly predicts learning behaviors. EVT suggests that when students possess both high expectations for success and high task value, they are more likely to exhibit positive behavioral responses, including learning initiative, persistence, and effort (Eccles and Wigfield, 2002). This interdependence between expectancy and value provides a more nuanced lens through which to interpret students' engagement in smart classrooms. This corresponds precisely to the learning engagement variable that this study focuses on.

This theoretical perspective helps understand the relationship between AI literacy, self-efficacy, learning satisfaction, and learning engagement. Currently, there are few studies on the relationship and influencing mechanisms between AI literacy and learning engagement. Based on EVT, this study introduces self-efficacy and learning satisfaction as two mediating variables to explore how AI literacy, as a cognitive background factor, can affect learning engagement in smart classrooms by enhancing students' self-efficacy (expectancy for success) and improving their learning satisfaction (task value). In practice, self-efficacy and learning satisfaction may also reinforce one another, as students who are confident often perceive greater value in the learning experience, while those who find learning meaningful tend to develop stronger efficacy beliefs.

This study aims to provide preliminary cross-sectional evidence on how AI literacy is associated with learning engagement through self-efficacy and learning satisfaction in smart classroom contexts. Unlike prior studies that have mainly examined direct relationships between digital or AI literacy and learning outcomes, the present study advances the literature by integrating self-efficacy and learning satisfaction within the Expectancy-Value Theory framework and testing their sequential mediating roles. In doing so, it provides a more specific explanation of how AI literacy may be translated into learning engagement through expectancy-related and value-related psychological processes in AI-enabled smart classrooms.

The central question addressed in this study is: how, and through which psychological pathways, does AI literacy relate to learning engagement in AI-enabled smart classroom environments? To answer this question, we propose and test the following hypotheses:

H1: AI literacy is positively associated with learning engagement in smart classrooms.

H2: Self-efficacy mediates the association between AI literacy and learning engagement.

H3: Learning satisfaction mediates the association between AI literacy and learning engagement.

H4: Self-efficacy and learning satisfaction serially mediate the association between AI literacy and learning engagement.

## 2 Literature review

### 2.1 AI literacy and learning engagement in smart classrooms

Against the backdrop of the rapid development in educational digitization and AI, AI literacy has become a key competency for measuring learners' ability to adapt and thrive in AI-driven environments. Ng (2012) expanded the concept of digital literacy into a comprehensive literacy framework encompassing technical skills, cognitive abilities, and social-emotional competencies. This framework emphasizes that digital literacy directly impacts learners' technological adaptability and autonomous learning in technology-intensive learning contexts. In recent years, research on AI literacy has gradually emerged. Long and Magerko (2020) define it as a set of core competencies required to understand, evaluate, and use AI systems, including algorithmic thinking, data literacy, and ethical awareness. Shao et al. (2024) found that adolescents' use of digital technology positively predicts their learning engagement, and that this relationship is strengthened through the mediating effect of flow experience, indicating that appropriate technology use can enhance students' immersion and participation in learning activities. Polat (2025) empirical research supports that AI literacy not only encompasses operational and application skills but also involves learners' critical understanding and innovative use of AI systems.

Learning engagement refers to learners' active participation in the learning process-behavioral, emotional, and cognitive (Fredricks et al., 2004), encompassing dimensions such as classroom participation, learning effort, and emotional engagement. Kahu and Nelson (2018) point out that learning engagement is not only influenced by individual background characteristics but also closely related to external factors such as the learning environment, instructional design, and social support. In smart classroom environments, AI and adaptive technologies make the explicit forms of learning engagement more diverse: interactive AI tools and adaptive learning can enhance student engagement; adaptive/personalized tasks and learning paths improve learning performance and satisfaction (Yaseen et al., 2025; Contrino et al., 2024). Existing research has explored the mechanisms linking digital literacy and technology literacy with learning engagement from the perspective of digital literacy or technology literacy. For example, Yaseen et al. (2025) found that digital literacy plays a moderating role in enhancing learning engagement in the use of AI-supported adaptive learning tools. Zakir et al. (2025) suggested that digital literacy significantly enhances students' engagement in informal learning environments. And the relationship between AI literacy and learning engagement has also been indirectly verified.

Yilmaz Soylu et al. (2026) found in their research on AI-driven assessment tools that learners with higher digital literacy levels showed significantly greater user satisfaction and participation frequency on the platform compared to the low-literacy group. This high-frequency participation is an important manifestation of learning engagement. Xu et al. (2025) conducted an empirical study of 1,079 Chinese college students and found that AI-driven personalized feedback significantly promoted learners' goal achievement and learning engagement, a mechanism that can be explained by self-determination theory. Bećirović et al. (2025) found that AI literacy is not only associated with academic performance but also closely linked to positive behavioral patterns in AI-enabled learning environments. Similarly, recent research in educational psychology and educational technology indicates that digital literacy enhances learners' self-efficacy for digital technology and perceived task value, which in turn positively influences learning engagement in technology-mediated learning environments (Yuan et al., 2025; Ai et al., 2025). A recent study examined how AI-enabled resources have been used to support self-directed learning (Younas et al., 2025b). However, that literature primarily concerns the affordances and use of AI resources rather than AI literacy as a learner-level capability. Direct evidence linking AI literacy to sustained engagement in smart classrooms therefore remains comparatively limited. In essence, while research directly exploring the relationship between AI literacy and learning engagement remains limited, existing research on digital literacy, technological literacy, and technology-enhanced learning provides a solid theoretical and empirical foundation for the potential connection between them. AI literacy can serve as the core competency foundation for learners in smart classroom environments. Through its associations with technology adaptability and learning enthusiasm, AI literacy can affect learners' learning engagement in smart classrooms.

### 2.2 The mediating role of self-efficacy

Self-efficacy, one of the core concepts of social cognitive theory (SCT), refers to an individual's belief in their ability to successfully complete tasks in specific situations (Bandura, 1997). This theory suggests that self-efficacy not only affects the level of effort and persistence of individuals in the learning process, but also influences their emotional regulation and strategy use (Schunk and DiBenedetto, 2020). In the context of technology-enhanced learning, learners' self-efficacy, as a key individual factor, can significantly predict their technology acceptance and willingness to use (Revythi and Tselios, 2019).

In recent years, numerous studies have verified the significant relationship between self-efficacy and learning engagement in the contexts of online learning, smart classrooms, and AI-powered learning. For example, in technology-enhanced learning contexts, technology self-efficacy and learning motivation/attitude jointly influence technology acceptance and continued use, thereby influencing learning engagement (Pan, 2020; Rosli and Saleh, 2023). Prior et al. (2016) point out that attitudes toward digital technology and digital literacy can promote students' self-efficacy, which in turn enhances students' various interactive behaviors in online learning (including peer engagement, LMS interaction, and convergent interaction). This echoes the chain path items in this study.

The enhancing effect of AI literacy on self-efficacy has been supported by multiple studies. The findings of Bećirović et al. (2025) also support that the technical understanding and practical application dimensions of AI literacy are significantly positively correlated with AI self-efficacy, while the critical evaluation dimension is negatively correlated. Higher AI self-efficacy is also associated with higher levels of AI output quality. Yilmaz Soylu et al. (2026) found that in AI-driven assessment systems, AI literacy not only improved students' proficiency in using the system, but also enhanced their confidence in tackling complex tasks. Similar findings have been found in the fields of digital literacy and technology literacy. Prior et al. (2016) pointed out that digital literacy can indirectly promote students' interactive behavior and learning persistence in online courses by enhancing their technology use self-efficacy. Tang and Chaw (2016) found in their blended learning research that technological self-efficacy played a significant mediating role between technological literacy and learning engagement.

Evidence also cautions against assuming that self-efficacy has a uniform role across AI-supported settings. In a study of AI integration, technological self-efficacy was not significantly associated with AI integration, whereas adaptive learning systems and digital interactive platforms showed positive associations (Younas et al., 2026). Theoretically, EVT suggests that self-efficacy is a core manifestation of expectancy for success (Eccles and Wigfield, 2002)^.^ In smart classroom environments, AI literacy can serve as an important background variable for learners, enhancing their confidence in completing learning tasks and thus stimulating higher levels of learning engagement. Combined with existing research, it can be inferred that self-efficacy may play a key mediating role between AI literacy and learning engagement. In a nutshell, self-efficacy is not only an important psychological factor influencing learning engagement, but also an important psychological mechanism by which AI literacy influences learning behavior.

### 2.3 The mediating role of learning satisfaction

Learning satisfaction is a comprehensive subjective evaluation of the learning process, interactive experience, system and course quality, and learning outcomes in technology-enhanced or online learning environments. It is considered a core emotional variable influencing e-learning success and quality. Existing research has systematically reviewed the key influencing factors from the dimensions of system, instructors, course, and learners, demonstrating that satisfaction is closely related to subsequent learning behaviors and outcomes (Sun et al., 2008). In the context of educational technology, three types of interaction (learner–instructor/content/learner) and Internet self-efficacy can significantly predict learning satisfaction in online courses (Kuo et al., 2014). Additionally, research grounded in technology acceptance and continuance models indicates that learners' satisfaction is a strong predictor of their continuance intention and subsequent use behavior in technology-mediated learning environments (Roca et al., 2006; Wu and Liu, 2023).

In the context of AI-driven smart classrooms, the latest evidence further supports the above pathway: adaptive/personalized tools can significantly improve learner satisfaction and learning performance (Contrino et al., 2024). Personalized feedback and interactive AI tools can significantly improve student engagement, and this effect is moderated by digital literacy (Yaseen et al., 2025). Separately, different interaction types have inconsistent effects on satisfaction, and the disaggregated evidence reinforces the validity of using satisfaction as a bridging variable (Wong and Chapman, 2023). At the instructional design level, the self-regulated flipped classroom helps improve learning performance, providing a methodological background for the relationship between “learning experience and engagement” (Lai and Hwang, 2016). Based on EVT, learners' positive assessments of task value and outcome expectations promote motivation and behavior, while learning satisfaction can be regarded as an emotional evaluation of this value and experience. Thus, it theoretically supports the mediating role of learning satisfaction in the “literacy influences engagement” pathway (Eccles and Wigfield, 2002; Sun et al., 2008). Recent studies further connect technology-related competence and affective experience to learning outcomes. Digital competence was positively associated with e-learning satisfaction in an emergency online-learning context (Younas et al., 2022), and a large cross-sectional study examined emotional learning in relation to student behavior, technological acceptance, mental wellbeing, cognitive engagement, and psychological resilience (Younas et al., 2025c).

### 2.4 Chain mediation of self-efficacy and learning satisfaction

In the context of smart classrooms, learners' behavioral engagement is often associated with both self-efficacy and learning satisfaction. Based on social cognitive theory, self-efficacy influences learners' judgments of task difficulty and controllability, as well as the resulting effort and persistence, and shapes subsequent learning experiences and behavioral performance through emotion regulation and strategy use (Bandura, 1997). In research on technology-enhanced learning and online learning, learning satisfaction is widely regarded as a key emotional mechanism linking cognitive beliefs and learning behavior: interaction quality and Internet self-efficacy can significantly enhance satisfaction (Kuo et al., 2014), and satisfaction itself is a key factor influencing online learning success (Sun et al., 2008).

Evidence from the information systems and educational technology fields, centered around the “belief-experience-behavior” sequential mechanism, supports that system/service quality and technology acceptance factors (such as perceived usefulness and perceived ease of use) consistently predict continued use intention and actual use behavior by increasing satisfaction. These behavioral indicators highly overlap with the behavioral aspects of learning engagement (Li and Phongsatha, 2022; Al-Hawamleh, 2024). Furthermore, research based on university samples has established a robust association between self-efficacy and learning engagement. Existing studies suggest that self-efficacy and learning engagement are closely related and may operate within different mediation or moderated-mediation pathways associated with academic outcomes (Alemayehu and Chen, 2023; Luo et al., 2023; Wang and Zhang, 2024).

In the specific context of AI empowerment, the empirical results also support this sequence: learners' interactions with generative AI can improve learning achievements through the chain mediation of self-efficacy and cognitive engagement, suggesting that initial sense of ability and success experiences will be translated into more positive experience evaluations and maintain a high level of engagement during the learning process involving AI tools (Liang et al., 2023). Based on the literature reviewed, it can be inferred that there exists a testable chain mechanism in smart classrooms: higher AI-related literacy enhances learners' self-efficacy, which in turn improves learning experience and increases learning satisfaction, ultimately leading to more persistent and proactive learning engagement. In conclusion, although research has not yet directly verified this complete pathway, existing studies all point to the conclusion that AI literacy indirectly affects learning engagement through the progressive effects of psychological beliefs and subjective experiences.

In short, existing studies suggest that AI literacy, self-efficacy, learning satisfaction, and learning engagement are closely interrelated in technology-enhanced learning environments. However, the complete chain of psychological mechanisms in AI-enabled smart classrooms has not yet been empirically tested. The present study extends prior research by moving beyond the examination of isolated direct or mediating relationships and testing an integrated sequential pathway from AI literacy to self-efficacy, learning satisfaction, and learning engagement. This gap highlights the need to examine whether AI literacy influences learning engagement through a sequential pathway involving self-efficacy and learning satisfaction, which forms the core focus of the present study (Figure 1).

**Figure 1:**
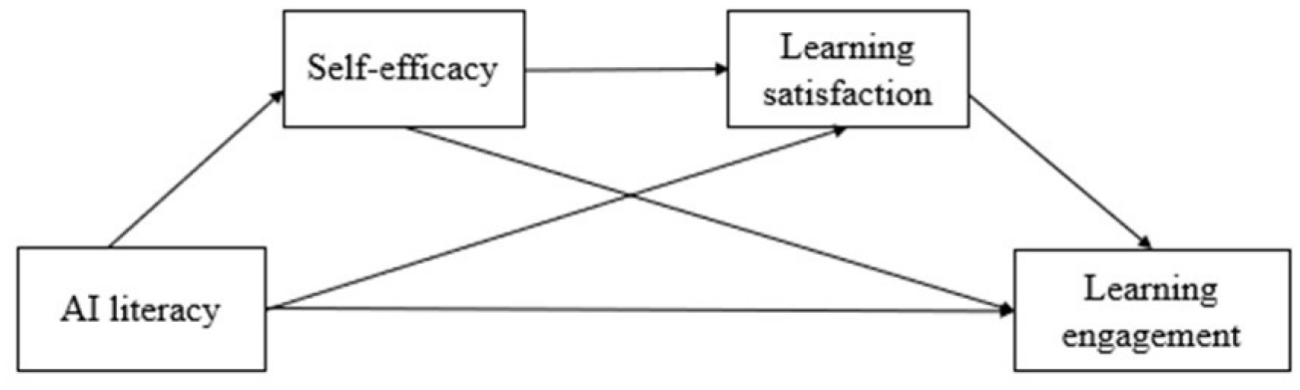
Chain mediation model.

## 3 Research methods

### 3.1 Data source

This exploratory cross-sectional study employed convenience sampling to recruit enrolled university students from 75 higher education institutions in China. At the institutional level, participating institutions were included through convenience-based access. They represented different regions and institutional categories, including Double First-Class universities, provincial key universities, ordinary undergraduate colleges, and junior colleges. This institutional coverage increased the descriptive breadth of the sample. The study protocol was approved by the Xiamen University Ethics Committee (No. XDYX202508K30).

At the participant level, the anonymous survey link was distributed to enrolled students at the participating institutions through Wenjuanxing (https://www.wjx.cn/). Eligible participants were students currently enrolled at one of these institutions who reviewed the informed-consent information and voluntarily completed the questionnaire. The consent page explained the study purpose, intended data use, anonymity of data collection, and voluntary nature of participation. Consent was indicated by voluntary submission, and no names or contact details were collected. Students who did not meet the enrollment criterion or did not voluntarily submit the questionnaire were not included.

A total of 3,000 questionnaires were collected. The submitted questionnaires were subsequently screened for completeness, response quality, and internal consistency. The recorded exclusion criteria included an unusually short response time, internally inconsistent responses, and the omission of multiple questionnaire items. These criteria were used to identify responses that might not provide sufficiently complete or coherent data for analysis. Following screening, 249 questionnaires were excluded. The final analytic sample comprised 2,751 valid questionnaires, corresponding to a retention rate of 91.70%. Table 1 presents the demographic and institutional characteristics of the final sample.

**Table 1:** Distribution of sample population characteristics (*N* = 2,751).

Variable name	Frequency	Percentage
**Gender**	
Male	1,148	41.7
Female	1,603	58.3
Grade	
First-year student (bachelor's/junior-college)	856	31.1
Second-year student (bachelor's/junior-college)	941	34.2
Third-year student (bachelor's/junior-college)	511	18.6
Fourth-year student (bachelor's/junior-college)	214	7.8
Master's student	207	7.5
PhD student	22	0.8
Place of residence	
Municipality	231	8.4
Prefecture-level city	695	25.3
County town	785	28.5
Township or rural area	1,040	37.8
Type of university	
Double first-class University	494	18.0
Provincial key university	662	24.1
Ordinary undergraduate college	1,057	38.4
Junior college	538	19.6

Percentages may not sum to 100.0% because values were rounded to one decimal place.

### 3.2 Research tools

This study employed four measurement instruments to assess AI literacy, learning engagement, self-efficacy, and learning satisfaction. All questionnaire items were rated using a five-point Likert response format ranging from 1 (strongly disagree) to 5 (strongly agree). When assessing the reliability, validity, and model fit of the measurement instruments, the statistical criteria presented in Table 2 were used.

**Table 2:** Criteria for judging statistical indicators.

Index indicator	Inspection objectives	Evaluation criteria
Cronbach's α	Scale internal consistency	≥0.70 (acceptable);
		≥0.80 (excellent)
KMO	Factor analysis applicability	>0.60 (acceptable);
		>0.80 (excellent)
Bartlett's test of sphericity	Variable correlation significance	*P* < 0.05
RMSEA	Model fitting error	≤ 0.08 (good);
		≤ 0.05 (excellent)
CFI/GFI/TLI	Overall model fit	≥0.90 (excellent);
		≥0.80 (acceptable)
*χ^2^*/df	Chi-square degrees of freedom ratio	< 3.00 (ideal); for large sample sizes, this indicator can be used as a reference and should be combined with RMSEA, CFI, GFI, NFI, and other indicators for a comprehensive assessment.

The above thresholds are general reference standards in the academic community, and their applicability may vary depending on the research background and sample size, and need to be reasonably explained according to the specific situation.

#### 3.2.1 AI literacy scale

AI literacy was assessed using the nine-item measurement instrument by Wang et al. (2023) which conceptualizes the construct through four competencies: cognition, application, evaluation, and ethics. This framework aligns well with the current study's focus on students' capacity to understand, utilize, and critically assess AI technologies in smart classrooms. Contextual adaptations were made to enhance relevance, with sample items including: “I can proficiently use AI applications or products to help me with my daily work” “I can choose the most suitable artificial intelligence application or product from many artificial intelligence applications or products for specific tasks” “I am always alert to the misuse of AI technology.” Responses were collected on a five-point Likert response format (1 = strongly disagree, 5 = strongly agree), with higher scores reflecting greater AI literacy. The scale exhibited excellent psychometric properties, showing high sampling adequacy (KMO = 0.957) and strong internal consistency (Cronbach's α = 0.956). A composite score was generated by averaging the nine items.

#### 3.2.2 Learning engagement scale

Learning engagement was measured using the four-item scale employed by Tu (2024). Tu (2024) reported that the four items were selected with reference to the smart-classroom learning engagement questionnaire reported by Zhang et al. (2019). The scale assesses students' overall learning engagement in smart classroom environments. Sample items include “In smart classrooms, I use various methods to process and understand the instructor's content” and “In smart classrooms, I am able to maintain a positive learning state.”

Responses were recorded on a five-point Likert scale ranging from 1 (strongly disagree) to 5 (strongly agree), with higher scores indicating higher levels of learning engagement. Tu (2024) reported good internal consistency for the four-item scale (Cronbach's α = 0.906) and provided exploratory factor-analytic evidence supporting its use (KMO = 0.828; Bartlett's χ^2^(6) = 648.975, *p* < 0.001; cumulative variance explained = 78.146%). In the present sample, the scale likewise demonstrated adequate factorability (KMO = 0.767) and high internal consistency (Cronbach's α = 0.934). In the confirmatory factor analysis of the full four-factor measurement model, the standardized loadings of the four learning-engagement items ranged from 0.892 to 0.922; composite reliability was 0.934 and the average variance extracted (AVE) was 0.826. A composite learning-engagement score was calculated by averaging the four item scores.

#### 3.2.3 Self-efficacy scale

Self-efficacy was assessed using the instrument developed by Pintrich and De Groot (1990) which measures students' confidence in their capacity to accomplish learning tasks and regulate their own learning processes. The construct has been extensively validated in educational settings and aligns closely with this study's focus on learners' perceived control and competence in AI-enhanced smart classrooms. We adapted the original scale to an eight-item measure contextualized to the current learning environment. Sample items include: “I am confident that I can understand the content of this course.” “I believe I have a good learning method to pass this course.” “I know that I am capable of learning the relevant study materials for this course.” All items were rated on a five-point Likert response format from 1 (strongly disagree) to 5 (strongly agree), with higher scores representing stronger self-efficacy. The scale exhibited excellent psychometric properties, showing high sampling adequacy (KMO = 0.928) and strong internal consistency (Cronbach's α= 0.951). A composite self-efficacy score was derived by averaging responses across the eight items.

#### 3.2.4 Learning satisfaction scale

Learning satisfaction was measured using the three-item measurement instrument developed by Hwang et al. (2014), originally designed to assess affective responses to technology-enhanced learning environments. The instrument, validated in contexts involving digital learning tools, provides a robust framework for evaluating student satisfaction in technology-supported settings–-closely aligning with this study's focus on smart classroom methodologies. Sample items include: “I like the way I learn in smart classrooms” “I believe that learning in smart classrooms can improve my learning effectiveness.” Participants responded using a five-point Likert response format ranging from 1 (strongly disagree) to 5 (strongly agree), with higher scores reflecting greater satisfaction. The scale demonstrated good factorability (KMO = 0.771) and high internal consistency (Cronbach's α = 0.943). A composite satisfaction score was generated by averaging the three items.

### 3.3 Data processing

SPSS26 and AMOS26 were used to conduct confirmatory factor analysis and correlation analysis on the data, testing the reliability of the questionnaire and the discriminant validity and correlation among the variables. Model 4 and Model 6 of the PROCESS macro developed by Hayes (2022) were used to analyze the mediation and serial indirect effects. PROCESS was selected because the constructs were represented by mean composite scores and the primary objective was to estimate prespecified direct and indirect associations using bootstrap confidence intervals. The significance of the mediation effect was tested using the percentile bootstrap method with 5,000 resamples. If the 95% confidence interval did not include 0, it was considered statistically significant (Zhou and Long, 2004). Before estimating the mediation model, multicollinearity among simultaneous predictors in the learning-engagement equation was assessed using variance inflation factors (VIFs), with 5 used as the threshold for serious concern.

## 4 Research results

### 4.1 Common method bias test

Given that all measures were self-reported and collected concurrently, we took both procedural and statistical steps to address potential common method bias. Procedurally, we ensured participant anonymity, used well-established scales with clear wording, and randomized the order of items where possible to reduce response biases. Before formal data analysis, the Harman single-factor method was used to test for common method bias, with the number of common factors set to 1, and AMOS26 was used to conduct confirmatory factor analysis, with the fit indices as follows: χ^2^/df= 70.912, *p* < 0.001, RMSEA = 0.159, SRMR = 0.058, GFI = 0.510, TLI = 0.747, CFI = 0.770. The single-factor model exhibited poor fit, which is consistent with the interpretation that severe common method bias is unlikely to exist in the study data. Because Harman's single-factor test provides only a preliminary diagnostic of common method bias, the result should be interpreted cautiously. Given that no marker variable, multi-source design, or latent method factor was incorporated, the poor fit of the single-factor model is taken only to indicate that the observed relationships were unlikely to be driven by a single dominant method factor (Podsakoff et al., 2003). Future studies could strengthen the assessment of common method bias by employing complementary procedural and statistical approaches.

### 4.2 Confirmatory and correlation analysis

First, AMOS26 was used for confirmatory factor analysis (CFA) to test the reliability and validity of the main variables. The CFA was conducted on the full four-factor measurement model (including all items across the four constructs) rather than on each scale in isolation. This integrated model demonstrated an excellent fit to the data: $\chi_{/\text{d}}^{2}$*f* = 12.353, *p* < 0.001, RMSEA = 0.064, SRMR = 0.015, GFI= 0.911, TLI= 0.959, CFI= 0.964. As shown in Table 3, the loadings of all factor items were greater than 0.60, and the *t*-values were significant at the 0.001 level, indicating that the structural validity of the scale was good. The AVE values of all factors were greater than 0.50, indicating good convergent validity of the scale. The square roots of the AVE values were all greater than the correlation coefficients between factors, indicating good discriminant validity of the scale. The Cronbach's α coefficients and composite reliability of all factors were greater than 0.70, indicating good reliability of the factors. In summary, the measurement instrument used in this study met acceptable standards in terms of reliability and validity (Schermelleh-Engel et al., 2003). Overall, the reported indices supported further analysis.

**Table 3:** Correlation coefficients, reliability and validity, and descriptive statistics of variables (*N* = 2,751).

Variable	M	SD	1	2	3	4	CR	AVE	CFA Loading range (mean)
1. AI literacy	3.664	0.654	0.844				0.957	0.713	0.716–0.897 (0.843)
2. Learning engagement	3.652	0.702	0.837 ^**^	0.909			0.934	0.826	0.892–0.922 (0.909)
3. Self-efficacy	3.512	0.696	0.668 ^**^	0.689 ^**^	0.843		0.951	0.711	0.803–0.881 (0.842)
4. Learning satisfaction	3.680	0.726	0.829 ^**^	0.887 ^**^	0.667 ^**^	0.913	0.938	0.834	0.897–0.924 (0.913)

^*^indicates *p* < 0.05, ^**^ indicates *p* < 0.01, ^***^ indicates *p* < 0.001, the same below. The diagonal values are the square roots of the average variance extracted (AVE). The table reports Fornell-Larcker evidence.

Correlation analysis of the main variables suggested that AI literacy, learning engagement, self-efficacy, and learning satisfaction were all significantly and positively correlated. As shown in Table 3, AI literacy was significantly positively correlated with learning engagement (*r* = 0.837, *p* < 0.01), self-efficacy (*r* = 0.668, *p* < 0.01), and learning satisfaction (*r* = 0.829, *p* < 0.01). Learning engagement was significantly positively correlated with self-efficacy (*r* = 0.689, *p* < 0.01) and learning satisfaction (*r* = 0.887, *p* < 0.01), and self-efficacy was significantly positively correlated with learning satisfaction (*r* = 0.667, *p* < 0.01). Given these high bivariate associations, particularly the correlations between learning satisfaction and learning engagement (*r* = 0.887) and between AI literacy and learning satisfaction (*r* = 0.829), multicollinearity diagnostics were examined before the mediation analysis. Multicollinearity concerns the predictors entered simultaneously in a regression equation; therefore, a high predictor-outcome correlation by itself does not establish multicollinearity. In the learning-engagement equation containing AI literacy, self-efficacy, and learning satisfaction as simultaneous predictors, the variance inflation factors were 1.952, 3.453, and 3.459, respectively, all below the criterion of 5 used in this study. Thus, although the high zero-order correlations warrant cautious interpretation, the VIF diagnostics do not indicate severe multicollinearity. Discriminant validity was also examined in Table 3. The square roots of AVE for learning engagement (0.909) and learning satisfaction (0.913) both exceeded their inter-construct correlation (*r* = 0.887); likewise, the square roots of AVE for AI literacy (0.844) and learning satisfaction (0.913) exceeded their correlation (*r* = 0.829). These results support discriminant validity under the criterion reported in this study and indicate that the constructs, while strongly related, should not be treated as statistically identical. Therefore, there was no serious multicollinearity problem in the data used in this study (Wen et al., 2018), making it suitable for further mediation test.

Before interpreting possible educational-level heterogeneity, measurement invariance of the four-factor measurement model was evaluated across different educational-level students. As shown in Table 4, the configural model yielded CFI = 0.925, TLI = 0.915, RMSEA = 0.094, and SRMR = 0.035. Although the RMSEA was above the conventional good-fit threshold, the incremental fit indices and SRMR provided an interpretable baseline for nested-model comparisons. Constraining factor loadings to equality produced only negligible changes (ΔCFI = −0.001, ΔRMSEA = −0.003, ΔSRMR = 0.009), supporting metric invariance. Further constraining item intercepts to equality did not materially worsen model fit (ΔCFI = 0.000, ΔRMSEA = −0.003, ΔSRMR = 0.002), supporting scalar invariance. Thus, the measures showed broad comparability across educational levels, providing a measurement basis for group comparisons.

**Table 4:** Measurement invariance across educational levels.

Model	χ^2^ (df)	CFI	TLI	RMSEA	SRMR	ΔCFI	ΔRMSEA	ΔSRMR
Configural	6,794.740 (1,344)	0.925	0.915	0.094	0.035	—	—	—
Metric	6,906.695 (1,439)	0.924	0.920	0.091	0.044	−0.001	−0.003	0.009
Scalar	7,005.636 (1,534)	0.924	0.925	0.088	0.046	0.000	−0.003	0.002

All chi-square tests were significant at *p* < 0.001. Δ values compare each model with the immediately preceding model. Scalar invariance was supported based on the small changes in CFI, RMSEA, and SRMR.

### 4.3 Hypothesis model testing

The present study employed the PROCESS macro developed by Hayes (2022), a well-established regression-based tool for estimating direct and indirect associations using composite scores and bootstrap confidence intervals. The analysis focused on associations among the four constructs and decomposed the total association into a direct effect and three prespecified indirect effects. PROCESS was therefore well suited to the study's association-focused analytical objectives. Given the cross-sectional design, the coefficients were interpreted as manifest-score associations rather than evidence of temporal ordering or causality.

First, AMOS26 was used to test the rationality of the hypothesis model. The results of the model fit indices are as follows: $\chi_{/\text{d}\text{f}=}^{2}$_16.141, p < 0.001, RMSEA = 0.074_, SRMR = 0.017, _GFI =_ _0.884, TLI =_ _0.945, CFI =__0.951_. While the χ^2^*/df* ratio is high and the GFI is slightly below the conventional threshold of 0.90, other indices such as TLI (0.945) and CFI (0.951) indicate an acceptable fit. It is important to note that the χ^2^*/df* statistic is highly sensitive to large sample sizes, which can lead to an inflated value and a significant *p*-value. Considering the overall pattern of indices, the structural equation model constructed based on the theoretical hypotheses is deemed reasonable and suitable for further hypothesis testing.

Subsequently, 5,000 percentile bootstrap resamples were drawn to estimate the 95% confidence interval (CI) of the indirect effects. The significance of the effect was tested by whether the 95% CI included 0. If the 95% CI did not include 0, it indicated a significant mediation effect (Fang and Zhang, 2012). The results of the chain mediation test model are shown in Table 5 and Figure 2.

**Table 5:** Regression equation of the chain mediation.

Outcome variable	Predictor variables	*R^2^*	*F*	β	*B*	*t*
Self-efficacy	Constant	0.447	2217.678^***^	—	0.905^***^	16.098
	AI literacy			0.668^***^	0.712^***^	47.092
Learning satisfaction	Constant	0.71	3370.470^***^	—	0.114^***^	2.577
	AI literacy			0.692^***^	0.768^***^	50.132
	Self-efficacy			0.205^***^	0.214^***^	14.874
Learning engagement	Constant	0.701	6436.615^***^	—	0.359^***^	8.622
	AI literacy			0.837^***^	0.899^***^	80.229
Learning engagement	Constant	0.827	4368.398^***^	—	0.082^***^	2.463
	AI literacy			0.283^***^	0.304^***^	19.161
	Self-efficacy			0.117^***^	0.118^***^	10.513
	Learning satisfaction			0.574^***^	0.555^***^	38.916

B denotes the unstandardized effect, and β denotes the completely standardized effect. The first learning-engagement equation reports the total effect; the second reports the direct effect after both mediators are included. ^***^indicates *p* < 0.001.

**Figure 2:**
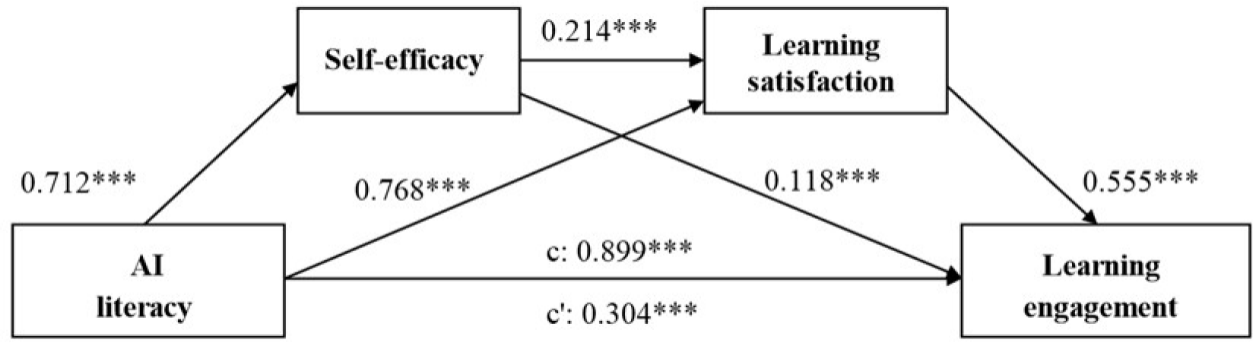
Chain mediation path coefficient. Values are unstandardized regression coefficients (B), c denotes the total effect, and c' denotes the direct effect after both mediators are included. ^***^indicates *p* < 0.001.

The corresponding completely standardized total, direct, and total indirect effect estimates were 0.837, 0.283, and 0.554, respectively. AI literacy was positively associated with learning engagement in the total-effect model [*B* = 0.899, SE = 0.011, 95% CI = (0.877, 0.921), *p* < 0.001], supporting H1. After both mediators were included, the direct association remained significant [B = 0.304, SE = 0.016, 95% CI = (0.273, 0.335), *p* < 0.001]. The total indirect effect was B = 0.595 [BootSE = 0.026, 95% bootstrap CI = (0.544, 0.644)] and accounted for 66.185% of the total effect, indicating partial mediation. At the displayed precision, the total effect equals the direct effect plus the total indirect effect (0.899 = 0.304 + 0.595). The model contained two mediators and three specific indirect paths (Table 6):

**Table 6:** Total, direct, and indirect effects of AI literacy on learning engagement.

Effect type	Unstandardized effect (B)	Standardized effect (β)	BootSE	LLCI/BootLLCI	ULCI/BootULCI	Effect proportion
Total effect	0.899	0.837	0.011	0.877	0.921	100.000%
Direct effect	0.304	0.283	0.016	0.273	0.335	33.815%
Total indirect effect	0.595	0.554	0.026	0.544	0.644	66.185%
Ind1	0.084	0.078	0.012	0.060	0.108	
Ind2	0.427	0.397	0.024	0.380	0.476	
Ind3	0.085	0.079	0.011	0.064	0.108	

B denotes the unstandardized effect, and β denotes the completely standardized effect. For the total and direct effects, model-based SEs and confidence intervals are reported; for the indirect effects, BootSEs and percentile bootstrap confidence intervals based on 5,000 resamples are reported. Percentages are calculated from the displayed three-decimal coefficients. The apparent 0.001 difference between the displayed total indirect effect and the sum of the three displayed specific indirect effects results from separately rounding each path estimate; the higher-precision PROCESS estimates are consistent. **Ind1**, AI literacy -> self-efficacy -> learning engagement; **Ind2**, AI literacy -> learning satisfaction -> learning engagement; **Ind3**, AI literacy -> self-efficacy -> learning satisfaction -> learning engagement.

(1) Ind1: AI literacy -> self-efficacy -> learning engagement [B = 0.084, BootSE = 0.012, 95% bootstrap CI = (0.060, 0.108)], supporting H2;

(2) Ind2: AI literacy -> learning satisfaction -> learning engagement [B = 0.427, BootSE = 0.024, 95% bootstrap CI = (0.380, 0.476)], supporting H3;

(3) Ind3: AI literacy -> self-efficacy -> learning satisfaction -> learning engagement [B = 0.085, BootSE = 0.011, 95% bootstrap CI = (0.064, 0.108)], supporting H4.

## 5 Discussions

Grounded in EVT, this study proposed and tested a hypothetical model linking AI literacy, self-efficacy, learning satisfaction, and learning engagement. Specifically, it examined the association between AI literacy and learning engagement and the serial indirect pathway through self-efficacy and learning satisfaction. The results showed that AI literacy was positively associated with learning engagement and that the serial indirect effect through self-efficacy and learning satisfaction was statistically significant. However, because all variables were measured concurrently, this serial pathway should be interpreted as a statistical decomposition of the observed associations.

### 5.1 The association between AI literacy and learning engagement

Data analysis results show that AI literacy was significantly and positively correlated with college students' learning engagement. According to EVT, individuals' level of engagement in the learning process depends not only on their judgment of the task value but also on their confidence in successfully completing it. Students with higher AI literacy tend to better understand and effectively use AI tools, thereby enhancing their sense of self-efficacy and task control during learning. This helps them maintain higher levels of learning motivation and sustained engagement both inside and outside the classroom. AI literacy can also expand students' learning strategies and information acquisition methods. This enables them to integrate resources and solve problems more efficiently during the learning process, thereby enhancing the depth and breadth of their learning. Empirical research has linked AI literacy both directly to learning engagement and indirectly to active participation through AI self-efficacy (He et al., 2025). Furthermore, a study by Bećirović et al. (2025) found that students with higher AI literacy significantly outperformed those with lower AI literacy in terms of confidence in using AI tools, output quality, and academic performance. Similarly, Shao et al. (2025) demonstrated that digital technology use positively influences English academic performance through the mediating roles of emotional intelligence and learning engagement, suggesting that technology-related competencies can enhance learning outcomes by fostering emotional and behavioral engagement. This advantage can further stimulate learning interest and engagement. These results are consistent with a positive association between AI literacy and learning engagement in smart classroom environments, and this study is consistent with existing research results.

Notably, even after accounting for the three significant mediating pathways, the direct effect of AI literacy on learning engagement remains substantial (*B* = 0.304, accounting for 33.815% of the total effect). This suggests that AI literacy retains a substantial association with students' engagement. One possible explanation is that AI literacy encompasses not only functional technical skills but also broader cognitive orientations, such as adaptability, digital confidence, and openness to innovation, which may be associated with proactive learning behavior. Additionally, in smart classroom environments, students with higher AI literacy may perceive AI-mediated learning as less cognitively demanding or anxiety-inducing, allowing them to sustain engagement even in the absence of increased satisfaction or efficacy. Future studies could further explore these direct psychological and behavioral mechanisms to unpack the remaining association with AI literacy.

### 5.2 The mediating role of self-efficacy

Research results indicate that self-efficacy plays a significant mediating role between AI literacy and learning engagement. Individuals' belief in their ability to complete tasks not only influences their effort and persistence but also shapes their perception of task value and emotional responses. Students with higher AI literacy adapt more quickly to AI-driven learning environments in smart classrooms, reducing uncertainty and anxiety caused by unfamiliarity with the technology, thereby forming higher expectations for success. This higher self-efficacy associated with AI literacy translates into learning engagement through at least three pathways: Primary, the goal-setting and strategy optimization effect: High self-efficacy encourages students to set more challenging goals and proactively adopt diverse strategies (such as AI-generated feedback and adaptive practice), thereby extending learning time and improving the quality of their engagement. Next, the emotion regulation effect: Students with high self-efficacy are more likely to view technical obstacles or difficult tasks as manageable challenges rather than insurmountable threats, reducing avoidance behaviors and maintaining stable engagement. Then, the feedback utilization effect: In AI-enhanced learning environments, high self-efficacy makes students more willing to receive and analyze immediate feedback, transforming it into a basis for strategy adjustments and ability improvement, forming a positive cycle of “feedback-adjustment-enhancement.” Notably, self-efficacy is enhanced not only by AI literacy itself, but also moderated by factors such as instructor and peer support, as well as past successful experiences. Insufficient social support may limit the translation of AI literacy into sustained engagement. Overall, the findings support an indirect association linking AI literacy, self-efficacy, and learning engagement. In particular, the positive association between AI literacy and self-efficacy is consistent with recent empirical research (Bewersdorff et al., 2025; Zhang et al., 2025).

### 5.3 The mediating role of learning satisfaction

The results indicate a significant indirect association between AI literacy and learning engagement through learning satisfaction. According to EVT, learning satisfaction reflects students' comprehensive emotional and cognitive evaluation of the learning process and its outcomes, encompassing the interest and practicality of the course, alignment with personal goals, and emotional experiences during the learning process. When students possess higher AI literacy, they are more effectively able to understand, apply, and evaluate AI tools in smart classrooms. This not only improves learning efficiency but also is associated with higher perceived value and emotional identification with the course, which corresponds to greater overall satisfaction.

This increased satisfaction, in turn, translates into higher learning engagement, primarily through three mechanisms: Primary, the emotional motivation effect: Positive learning experiences stimulate students' interest and enjoyment in learning, making them more willing to invest time and energy in in-depth learning. For example, Cigdem and Ozturk (2016) found that in technology-enabled learning management systems (LMS), the interactivity and multimedia features of the system significantly increased learners' perceived satisfaction. This heightened satisfaction, in turn, promoted sustained engagement and commitment. Sánchez and Hueros (2010) also noted that perceived satisfaction indirectly influenced learning motivation and behavioral engagement by increasing perceived usefulness and ease of use. Next, the goal-driven effect: highly satisfied students are more likely to perceive course content as highly aligned with their personal development goals, leading them to set more proactive and sustained learning plans. Kuo et al. (2013)8 found that in online learning environments, learning satisfaction is not only positively correlated with learning motivation but also enhances learning outcomes through goal alignment and a sense of belonging. A review by Lee and Choi (2011) further demonstrated that satisfaction plays an important role in maintaining learners' long-term engagement and reducing dropout rates. Then, the trust and platform stickiness effect: In smart classroom environments, satisfaction with platform functionality, feedback mechanisms, and AI tools increases students' trust and reliance on the system, thereby maintaining long-term learning engagement.

Learning satisfaction not only impacts emotional and motivational aspects but may also indirectly influence learning engagement through a sense of social belonging. Smart classrooms often include collaborative learning components. When students are satisfied with the effective support and friendly learning atmosphere provided by AI tools, they are more willing to participate in group interactions and knowledge sharing, further enhancing their behavioral engagement and learning outcomes (Alqurashi, 2016). In summary, the mediating role of learning satisfaction goes beyond a single emotional response to encompass the combined effects of cognitive evaluation, goal fit, trust, and social belonging. This study's findings are consistent with numerous empirical studies demonstrating that, in technology-supported learning environments, positive learning experiences and high satisfaction can significantly promote students' comprehensive engagement at the cognitive, emotional, and behavioral levels. Numerically, the estimated specific indirect effect through learning satisfaction alone [Ind2 = 0.427, 95% CI = (0.380, 0.476)] was larger than the estimated indirect effects through self-efficacy alone and through the serial pathway. This descriptive pattern suggests that learning satisfaction may be a particularly salient pathway in the present sample. However, the numerical differences should therefore be interpreted descriptively and do not establish that the indirect pathways differ statistically.

From a theoretical standpoint, this finding is consistent with expanded interpretations of Expectancy-Value Theory (EVT), which suggest that value judgments and emotional responses may exert a stronger influence on sustained behavior than expectancy beliefs. In the context of technology-enhanced smart classrooms, improving students' satisfaction–-by optimizing system usability, enhancing content relevance, and strengthening emotional support and feedback mechanisms–-may be a relevant strategy for supporting long-term learning engagement.

### 5.4 The mediating role of self-efficacy and learning satisfaction

Research results indicate that AI literacy was indirectly associated with college students' learning engagement through the serial pathway involving self-efficacy and learning satisfaction. When students possess higher AI literacy, they are not only more proficient in technology use and learning strategies, but also demonstrate greater confidence and a sense of control over learning tasks, which is associated with higher self-efficacy. On this basis, this positive self-perception is associated with higher levels of learning satisfaction, including increased interest in the course content, recognition of its practical value, and positive emotional experiences during the learning process. High levels of learning satisfaction further translate into students' sustained engagement and deep involvement both inside and outside the classroom, forming the proposed chain of “AI literacy-self-efficacy-learning satisfaction-learning engagement.” This ordering should be interpreted as associational rather than temporal.

Students with high self-efficacy are more likely to adopt positive coping strategies when faced with learning challenges, maintain motivation and regulate emotions, thereby laying the foundation for improved satisfaction. Learning satisfaction is a comprehensive emotional and cognitive evaluation of the learning process and outcomes, encompassing aspects such as course interest, practicality, goal fit, and emotional experience. In the context of smart classrooms, increased satisfaction not only means greater emotional engagement, but also reflects students' recognition and trust in the teaching model and the application of AI tools. This emotional identification ultimately translates into more sustained learning behavior engagement.

Existing empirical research has provided support for this chain mediation mechanism. Luo et al. (2023) found a significant positive correlation between academic self-efficacy, learning engagement, and academic achievement among Chinese college students, which is consistent with this study. Chiu (2021) also found in a qualitative analysis of K-12 online learning contexts that the combination of self-efficacy and satisfaction significantly affected students' continued engagement and learning stickiness during the epidemic. These studies are consistent with the results of this study, indicating that in technology-supported learning environments, the synergistic effect of cognitive variables (self-efficacy) and emotional variables (learning satisfaction) is an important mechanism for promoting learning engagement.

### 5.5 Research implications

Based on EVT, this study examines an associative theoretical model linking AI literacy with college students' learning engagement through the chain mediation effect of self-efficacy and learning satisfaction. This research examines the association between AI literacy and learning engagement through a cognitive-emotional pathway in the context of smart classrooms. The research findings not only expand theoretical perspectives on the relationship between AI literacy and learning behavior but also provide practical references for smart classroom construction, digital literacy education, and learning motivation cultivation.

#### 5.5.1 Theoretical implications

This study deepens the understanding of how AI literacy may relate to learning behavior by elucidating a cognitive-emotional-behavioral mechanism linking AI literacy, self-efficacy, learning satisfaction, and engagement. It extends the theoretical framework of Expectancy-Value Theory (EVT) in technology-enhanced learning contexts by providing cross-sectional evidence consistent with both the independent and chain mediation roles of expectancy (self-efficacy) and value (learning satisfaction).

At the descriptive level, the estimated indirect effect through learning satisfaction (Ind2 = 0.427) was numerically larger than that through self-efficacy (Ind1 = 0.084) or the serial pathway (Ind3 = 0.085). Because no pairwise inferential contrasts were conducted, this pattern should not be interpreted as demonstrating statistically different pathway magnitudes. It may nevertheless suggest that, in AI-mediated learning settings, perceived usefulness, emotional resonance, and relevance warrant particular attention alongside confidence when examining sustained learning behaviors. This interpretation remains tentative because the cross-sectional design does not establish causal priority between value-related and expectancy-related processes.

Moreover, the substantial direct effect of AI literacy (*B* = 0.304), even after accounting for mediators, suggests that current motivation models may overlook important components of digital competence. This residual association may reflect factors such as students' digital identity, perceived control over AI tools, or openness to innovation–-dimensions not traditionally addressed in EVT. As such, our findings provide evidence relevant to EVT in a new context while also identifying constructs for future examination. Future theoretical models could benefit from integrating broader constructs of digital adaptability and affective–motivational readiness to better account for learner behavior in technology-rich educational environments.

#### 5.5.2 Practical implications

It is essential for colleges and universities to incorporate AI literacy cultivation into the core of their talent development systems. Through systematic courses and practical training, they should enhance students' ability to understand, evaluate, and apply AI tools. Such training should address not only cognitive understanding and practical application, but also the critical evaluation and ethical use of AI-generated information. This may strengthen their sense of task control and success expectations in learning, which lays a solid foundation for sustained engagement. Course design should emphasize the deep integration of AI technology and professional knowledge and create authentic application scenarios to guide students in actively using AI tools in problem-solving, data analysis, and knowledge construction, which may enhance the contextual relevance and value of learning.

#### 5.5.3 Pedagogical implications

It is also important to systematically enhance students' self-efficacy, particularly in AI-enabled learning environments. Instructors can help students accumulate and master experience and reduce uncertainty and anxiety of technology use through tiered task design, immediate feedback, and positive motivation. During this process, establishing a supportive classroom atmosphere and peer support mechanisms may be associated with greater student confidence and stronger coping skills, which may be associated with learning motivation and sustained engagement.

In addition, significant attention should be paid to cultivating and improving learning satisfaction. Colleges and instructors should optimize the interactivity, feedback mechanisms, and resource provision of smart classroom platforms, enabling students to gain positive emotional experiences and value recognition during the learning process. At the same time, it is important to ensure that the learning content is highly aligned with students' interests, career plans, and development goals, while increasing the interest and challenge of the courses. By creating an open, collaborative, and inclusive learning environment, students' attachment to the learning platform and teaching model can be enhanced, thereby maintaining long-term behavioral engagement.

#### 5.5.4 Integrated implications for smart classroom design

Finally, the observed pattern suggests that technological literacy is associated with learning engagement alongside cognitive (self-efficacy) and emotional (learning satisfaction) variables. Therefore, colleges and universities should adopt a comprehensive strategy in the construction of smart classrooms. This involves organically integrating technical training, psychological support, and emotional motivation to build a learning ecosystem with cognitive-emotional-behavioral synergistic effects. Such an ecosystem may support students' engagement depth and learning outcomes in smart classrooms.

### 5.6 Limitations

The limitations of this study are mainly reflected in the following aspects.

First, the research design is limited by its cross-sectional nature. This study uses cross-sectional data, which elucidate the associative pathways between AI literacy, psychological mechanisms, and learning engagement. Because all variables were measured at the same time, the mediation model cannot disentangle whether AI literacy promotes engagement, whether more engaged students develop stronger perceived AI literacy and satisfaction, or whether both are shaped by unmeasured third variables such as general motivation, technology readiness, GPA, or prior academic achievement. Therefore, all relationships reported should be interpreted as associations rather than causal effects. The design also cannot capture the dynamic evolution of learning engagement in smart classroom environments.

Second, the sampling method may restrict the generalizability of findings. This study adopted convenience sampling to collect data, a non-probability sampling method commonly used in large-scale surveys (*N* = 2,751) for its practicality. While this approach facilitated data collection across 75 colleges and universities in China, it does not guarantee random selection of participants from the broader population of higher education students. This, combined with the over-representation of undergraduate students and under-representation of graduate and international learners, further limits the external validity of the study's conclusions. In addition, convenience-based institutional access and voluntary response may have produced self-selection and unequal participation opportunities across institutions. Accordingly, the breadth of institutional coverage should be understood as descriptive coverage.

Third, the measurement instruments were not specifically developed for AI-enabled smart classroom contexts. Although the scales demonstrated satisfactory reliability and validity, some items remained oriented toward general technology-supported learning. They may not fully capture context-specific experiences such as intelligent feedback, human–computer collaborative teaching, and personalized or adaptive learning. Consequently, the measures may capture the broader dimensions of AI literacy and self-efficacy while providing less precise assessments of their manifestations in smart classrooms. This may limit the specificity with which the relationships in the proposed model can be interpreted. Relatedly, all constructs were assessed through self-report measures administered in a single questionnaire at one time point. These shared measurement conditions may have contributed to the observed covariation and should be considered when interpreting the high correlations. The one-factor test provided an initial assessment of common method bias, and its poor fit suggested that a single dominant method factor was unlikely to explain the observed relationships. Given the strong associations among several constructs, further item-level validation would be beneficial. Future studies could refine the contextual wording of the items and report HTMT with a bootstrap confidence interval (Henseler et al., 2015).

Fourth, the study did not consider moderating mechanisms and contextual heterogeneity. The study focuses on the main effects of chain mediation pathways and does not include moderating variables at the individual level (such as technology anxiety and learning styles) or the environmental level (such as instructors' AI teaching ability, quality of classroom interaction, and college technical support). This fails to reveal the differential manifestations of the influencing mechanisms under different conditions. Relatedly, although scalar measurement invariance across educational levels was supported, residual heterogeneity by educational level may remain, and the pooled indirect effects should be interpreted as overall average associations rather than as evidence of identical mechanisms across groups. Future studies with more balanced educational-level samples should directly test structural path invariance and compare group-specific indirect effects.

Fifth, the analytical approach does not account for the nested data structure or latent nature of constructs. The dataset in this study is hierarchical, with students (*N* = 2,751) nested within 75 higher education institutions. Furthermore, our constructs (AI literacy, self-efficacy, etc.) are conceptualized as latent variables. The PROCESS macro assumes independent observations and uses manifest composite scores. If students within the same institution are correlated, standard errors may be underestimated and *p* values and confidence intervals may be too optimistic. Because ICCs, design effects, and cluster-robust or multilevel estimates were not obtained, the extent of this risk cannot be quantified. Although the findings offer initial evidence of individual-level relationships, the effect sizes may have limitations.

### 5.7 Recommendations for future research

To address these limitations, future research can be carried out in the following directions.

First, future studies should optimize sampling methods to enhance generalizability. To mitigate the limitations of convenience sampling, probability sampling methods could be adopted. This would ensure a more representative selection of participants across different genders, academic disciplines, educational stages, and cultural backgrounds thereby improving the external validity of research findings.

Second, future studies could adopt mixed research methods to capture the dynamic process of learning engagement; design quasi-experimental studies to enhance the reliability of causal inferences and ensure that the research findings have a stronger scientific basis. In addition, the scope of the study could be expanded by increasing the sample size, including more educational stages and international learners, to enhance the generalizability of the research findings.

Third, researchers should refine the measurement of AI literacy by distinguishing its dimensions and analyzing their unique impact pathways. Concurrently, measurement tools should be optimized for smart classroom contexts. Future item-level validation should also report HTMT with a bootstrap confidence interval and test measurement invariance across relevant groups. Moreover, future research could expand the research variables by introducing moderating variables to examine boundary conditions, thereby revealing the differential manifestations of the influencing mechanisms under different conditions.

Fourth, future work should deepen theoretical integration by combining frameworks such as self-determination theory (SDT) or the technology acceptance model (TAM) to explore how AI literacy affects learning engagement through autonomy needs or perceived usefulness. This would help construct a multi-theoretical integration framework to more comprehensively understand the mechanism of AI literacy in smart classroom environments.

Moreover, to address the methodological limitation of this study, future research should employ analytical techniques that are appropriate for nested data and latent constructs. For instance, multilevel structural equation modeling (MSEM) could be utilized to simultaneously account for student-level predictors and institution-level contextual factors. Specifically, researchers might incorporate college-level variables–-such as the extent of AI teaching resource allocation, faculty AI competency training, or institutional support for smart classroom infrastructure–-as cross-level moderators to examine how these environmental conditions shape the strength of the proposed mediation pathways.

## 6 Conclusion

This study employed an exploratory cross-sectional design to investigate the associations between AI literacy and college students' learning engagement in AI-enabled smart classrooms, as well as the psychological mechanisms through whi**c**h such associations might emerge. Grounded in Expectancy–Value Theory (EVT), we proposed a chain mediation framework involving self-efficacy and learning satisfaction and explored their potential roles as cognitive and affective pathways.

The findings provide preliminary evidence that higher levels of AI literacy are associated with greater learning engagement among college students. In addition, the analyses suggest that self-efficacy and learning satisfaction may each serve as independent mediators, and may also operate sequentially as a potential chain pathway. These associative patterns point to several promising mechanisms–-such as enhanced confidence in handling AI-supported tasks and more positive emotional evaluations of the learning process–-that may help explain how students navigate technology-rich learning environments. Given the exploratory nature of the study, these results should not be interpreted as establishing causal effects. Instead, they offer an initial understanding of possible psychological routes through which AI literacy may correspond with engagement in smart classrooms. In light of the literature reviewed, the findings also extend EVT into AI-mediated learning contexts by highlighting the joint functioning of expectancy (self-efficacy) and value-related appraisals (learning satisfaction) in understanding students' learning behaviors.

Several tentative implications emerge from this exploration. Enhancing AI literacy may represent one potential avenue to support student confidence and facilitate positive learning experiences. At the same time, instructional design that strengthens students' perceptions of relevance, usefulness, and emotional satisfaction may help sustain engagement in technology-rich classrooms. These insights are not definitive prescriptions but suggest directions for future pedagogical and technological development. Overall, this study provides a preliminary theoretical and empirical groundwork for understanding the cognitive–emotional mechanisms that may connect AI literacy with student engagement. Longitudinal, experimental, and multilevel designs will be essential in future research to validate these pathways and further clarify how learners adapt to increasingly AI-integrated educational environments.

## Data Availability

The raw data supporting the conclusions of this article will be made available by the authors, without undue reservation.
